# Supplementary material for: A Retrospective Analysis of Endovascular Stent Insertion for Malignant Superior Vena Cava Obstruction, Focusing on Anticoagulation Practices
Source: Curr Oncol. 2025 Oct 27;32(11):601. doi: 10.3390/curroncol32110601 (PMC12651178; doi:10.3390/curroncol32110601)
Supplement: Supplementary file 1 [file curroncol-32-00601-s001.zip › curroncol-3829965-supplementary.pdf]

Supplementary Materials

Table S1. Patient characteristics, stent details, complications, anticoagulation therapy and survival outcomes.

Number of Patients	Age at Diagnosis (Range)	Primary Malignancy Origin	Type of Stent	Stent Complications	Known Thrombotic/Bleeding Complications	Anticoagulation Plan at Discharge	Change in Therapy (Why)	Time from Stent to Death (Days)
1	55-69	Lung	Uncovered	-	-	Tx LMWH[1]	-	50
2	60-64	Lung	Uncovered	Stent thrombosis	-	Tx LMWH	Additional therapy (thrombosis)	41
3	55-59	Lung	Uncovered	-	-	Tx LMWH and 75mg Aspirin	-	330
4	50-54	Lung	Two stents	-	-	Px LMWH[2]	-	33
5	70-74	Lung	Uncovered	-	-	Tx LMWH	-	21
6	50-54	Lung	Two stents	Stent stenosis/occlusion	Pulmonary embolism and Subclavian thrombosis	Aspirin	Change in agent (thrombosis)	385
7	60-64	Breast	Uncovered	-	Deep vein thrombosis and Internal Jugular vein thrombosis	Tx LMWH	-	604
8	55-59	Lung	Two stents	Stent thrombosis	Subclavian thrombosis	Px LMWH	Dose escalation (thrombosis)	163
9	40-44	Breast	Uncovered	-	-	Tx LMWH	-	72
10	75-79	Lung	Uncovered	Stent thrombosis	-	Tx LMWH	-	147
11	80-84	Lung	Uncovered	-	-	Tx LMWH	-	28
12	50-54	Lung	Uncovered	-	-	Tx LMWH	-	33
13	55-59	Lung	Two stents	-	-	Px LMWH and 75mg Aspirin	-	13
14	70-74	Lung	Uncovered	Stent thrombosis	-	Tx LMWH	Dose reduction (preference after treatment for thrombosis)	150
15	50-54	Lung	Uncovered	Stent thrombosis	-	Tx LMWH	Change in therapy (preference)	82
16	70-74	Lung	Uncovered	-	-	Tx LMWH[1]	-	30

17	70-74	Lung	Uncovered	-	Pulmonary embolism	Nil	Change in therapy (thrombosis and preference) x2	613
18	50-54	Breast	Uncovered	Stent thrombosis	-	Tx LMWH	-	28
19	65-69	Lung	Uncovered	-	-	Tx LMWH	-	179
20	75-79	Lung	Uncovered	-	Pulmonary embolism	Nil	Additional therapy (thrombosis)	50
21	75-79	Lung	Two stents	-	-	NA[3]	-	10
22	25-29	Lung	Uncovered	-	-	Nil	-	243
23	80-84	Breast	Uncovered	-	-	Apixaban 2.5mg BD[4]	-	51
24	65-69	Thyroid	Two stents	-	-	Tx LMWH	-	264
25	55-59	Upper GI	Uncovered	-	-	Tx LMWH	-	6
26	50-54	Lung	Uncovered	-	-	Px LMWH	-	283
27	55-59	Lung	Uncovered	-	-	Tx LMWH	Change in agent (preference)	127
28	55-59	Lung	Two stents	-	-	Px LMWH and 75mg Aspirin	-	66
29	65-69	Lung	Covered	-	-	Px LMWH	-	62
30	70-74	Lung	Uncovered	-	-	Tx LMWH	-	40
31	55-59	Lung	Two stents	Stent stenosis/occlusion	Jugular vein thrombosis	Tx LMWH	Change in agent (thrombosis)	226
32	55-59	Breast	Two stents	Stent stenosis/occlusion	-	NA	-	2
33	60-64	Lung	Uncovered	Stent thrombosis	-	Tx LMWH	Change in agent (preference)	159
34	65-69	Lung	Covered	-	-	Tx LMWH	Change in agent (preference)	63
35	70-74	Lung	Two stents	-	-	Nil	-	231
36	60-64	Lung	Covered	-	-	Tx LMWH	-	90
37	45-49	Lung	Two stents	-	-	Tx LMWH	-	78
38	70-74	Lung	Covered	-	-	Tx LMWH	-	71
39	55-59	Lung	Uncovered	-	Pulmonary embolism	NA	-	7
40	75-79	Lung	Covered	-	-	Px LMWH	Change in dose x2 (Escalation and reduction)	87

41	75-79	Lung	Uncovered	-	Traumatic hemorrhagic bursitis	Px LMWH and 75mg Aspirin	Change in dose x2 (bleeding, re-establishment)	73
42	70-74	Lung	Two stents	-	-	Tx LMWH	Change in agent (preference)	28
43	55-59	Lung	Covered	-	-	Tx LMWH	-	5
44	40-44	Breast	Covered	Stent stenosis/occlusion	-	Tx LMWH	Change in dose x2 (escalation and reduction)	89
45	40-44	Lung	Covered	-	-	NA	-	3
46	40-44	Breast	Uncovered	-	Deep vein thrombosis	Tx LMWH	Change in agent (preference)	114
47	45-49	Lung	Covered	SVCO[5] recurrence	-	DAPT[6]	-	41
48	55-59	Lung	Covered	-	Pulmonary embolism	Tx LMWH	Change in agent x2 (preference and absorption)	93
49	70-74	Lung	Two stents	-	-	Apixaban 5mg BD	-	27

1Tx LMWH: Treatment dose low molecular weight heparin. 2Px LMWH: Prophylactic dose low molecular weight heparin. 3NA: Not applicable/available. 4BD: Twice daily. 5DAPT: Dual anti-platelet therapy. 6SVCO: Superior vena cava obstruction.
